# HLA-G Molecules in Autoimmune Diseases and Infections

**DOI:** 10.3389/fimmu.2014.00592

**Published:** 2014-11-18

**Authors:** Roberta Rizzo, Daria Bortolotti, Silvia Bolzani, Enrico Fainardi

**Affiliations:** ^1^Section of Microbiology and Medical Genetics, Department of Medical Sciences, University of Ferrara, Ferrara, Italy; ^2^Neuroradiology Unit, Department of Neurosciences and Rehabilitation, Azienda Ospedaliera-Universitaria Arcispedale S. Anna, Ferrara, Italy

**Keywords:** HLA-G, inflammation, autoimmunity, infection, regulation

## Abstract

Human leukocyte antigen (HLA)-G molecule, a non-classical HLA-Ib molecule, is less polymorphic when compared to classical HLA class I molecules. Human leukocyte antigen-G (HLA-G) was first detected on cytotrophoblast cells at the feto-maternal interface but its expression is prevalent during viral infections and several autoimmune diseases. *HLA-G* gene is characterized by polymorphisms at the 3′ un-translated region and 5′ upstream regulatory region that regulate its expression and are associated with autoimmune diseases and viral infection susceptibility, creating an unbalanced and pathologic environment. This review focuses on the role of HLA-G genetic polymorphisms, mRNA, and protein expression in autoimmune conditions and viral infections.

## Introduction

**Figure 1:**
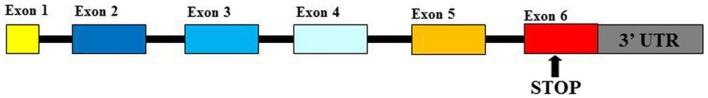
**Human leukocyte antigen-G gene**. UTR, un-translated region.

Human Leukocyte Antigen-G (HLA-G) is a functional molecule belonging to class Ib human leukocyte antigens (HLA) characterized by a non-covalent link between β_2_-microglobulin (β2m) and glycoprotein heavy chain. The gene is located within Major Histocompatibility Complex (MHC) locus on chromosome 6 (1, 2). HLA-G products show some peculiar features for which they are considered as non-classical HLA-I antigens: (1) the limitation of their allelic polymorphism (3); (2) the expression of seven isoforms represented by four membrane-bound (G1, G2, G3, and G4) and three soluble (G5, G6, and G7) proteins (4); and (3) the restriction of their tissue distribution (5). Polymorphisms at the 5′ upstream regulatory region and at the 3′ UTR of the *HLA-G* gene play an important role in the regulation of HLA-G production (6). Mainly, two polymorphisms at the 3′ UTR: a deletion/insertion (*DEL/INS*) of 14 base pairs (*14bp*) polymorphism (rs371194629) and a *C* > *G* single-nucleotide polymorphism (SNP) at the +*3142bp* position (rs1063320) (7) (Figure 1) are able to affect mRNA stability *in vivo* and protein production and implicated in pathological conditions: *14bpINS* allele is associated with mRNA instability (8, 9); +*3142G* allele creates a binding site for three microRNAs (miRNAs) (*miR-148a, miR-148b*, and *miR-152*) reducing soluble protein production (10). These observations suggest that *14bpINS/INS* and +*3142G/G* genotypes are associated with a lower HLA-G production than *14bpDEL/INS* and *DEL/DEL*, +*3142C/G*, and *C/C* genotypes (8, 10).

**Figure 2:**
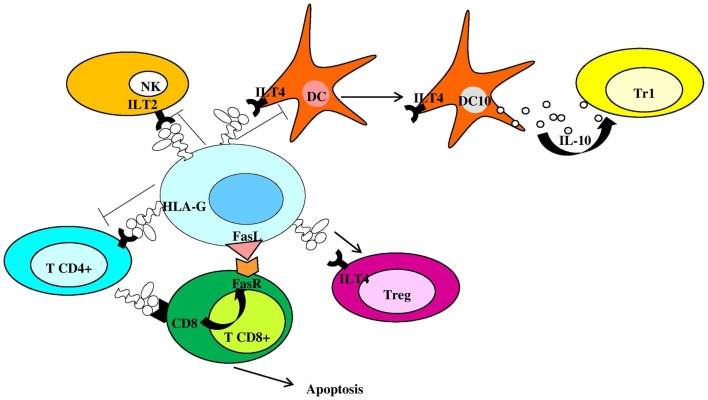
**Human leukocyte antigen-G is an anti-inflammatory molecule inhibiting and controlling immune cell activation**. NK, natural killer cells; Tr1, type 1 regulatory T cells; DC, dendritic cell; Treg, regulatory T cell; FasR, Fas receptor; DC10, IL-10-differentiated dendritic cells.

Membrane-bound HLA-G1 and soluble HLA-G5 (HLA-G5) represent the mainly expressed and investigated HLA-G isoforms (1) and are currently supposed to be the most important and functional isoforms (11). However, while HLA-G5 molecules are actively secreted as soluble isoforms, HLA-G1 proteins could be released by proteolytic shedding from cell surface (sHLA-G1) via matrix metalloproteinase-2 (MMP-2) (12–16). HLA-G can exist as β2m-associated and -free monomers (17, 18) and as disulfide-linked dimers or multimers (17, 19, 20). HLA-G disulfide-linked dimers are linked by disulfide bonds between two cysteine residues at position 42 of the HLA-G alpha-1 domain (19–21) and present higher affinity for ILT-2 and ILT-4 receptors compared to monomers (22, 23). Placental trophoblast cells (24), thymus (25), cornea (26), nail matrix (27), pancreas (28), erythroid, and endothelial precursors (29) present a physiological expression of HLA-G molecules. However, HLA-G can be ectopically expressed also on monocytes (30), in transplantation, tumors, viral infections, and autoimmune diseases (1, 2). HLA-G antigens are currently considered as immune-modulatory molecules due to their role in preserving immune tolerance at the feto-maternal interface (31), promoting graft tolerance (32), reducing inflammatory and immune responses (33), favoring tumors (34), and virus infection via immune escape (35). Both membrane-bound and soluble HLA-G antigens exert their immune-suppressive properties: (a) inhibiting the activity and inducing apoptosis of cytotoxic CD8^+^ T cells and NK cells (36–38); (b) inhibiting the proliferation of CD4^+^ T cells that are shifted to an immune-suppressive profile (39, 40); (c) inhibiting antigen-presenting cells and B cell differentiation (41, 42); (d) inducing a Th2 polarization (43); and (e) inducing regulatory T cells (44) and Interleukin (IL)-10 secreting dendritic cells (DC10) (45) (Figure 2). The interactions between HLA-G proteins and their specific inhibitory receptors ILT-2 (LILRB1/CD85j), ILT-4 (LILRB2/CD85d), and KIR2DL4 (CD158d) expressed by immune cells (46) account for the effects of these molecules on immune cells.

Moreover, HLA-G expression is up-regulated by the secretion of anti-inflammatory cytokines such as IL-10 which, in its turn, is enhanced by HLA-G (30). For these reasons, the implication of HLA-G molecules in inflammatory, immune-mediated, and infective conditions has been investigated (47, 48). The knowledge of the interactions between HLA-G molecules and immune mechanisms and their implication in pathological conditions may assist in improving our knowledge on the mechanisms at the basis of several autoimmune diseases and viral infections.

## HLA-G and Gastrointestinal Diseases

Celiac disease is a gluten sensitivity, which induces an inflammation that damages the villi in the small intestine of genetically predisposed subjects. Both genetic and environmental factors contribute to the development of celiac disease (CD). Torres and coauthors (49) have shown the presence of HLA-G in biopsies from celiac patients and have observed higher sHLA-G amounts in comparison with control subjects. The evaluation of the *14bp INS/DEL* polymorphism in a group of 522 celiac patients (50), subdivided accordingly with the presence of HLA-DQ2 molecule, encoded by DQA1*05/DQB1*02 genes, has demonstrated an increased frequency of the *14bp INS/INS* genotype in comparison with controls. These data suggest that the *14bp INS* allele may increase the risk of gut inflammation, most likely leading to chronicity. Ulcerative colitis (UC) and Crohn’s disease are characterized by a different sHLA-G expression pattern (51) by peripheral blood mononuclear cells. Non-activated peripheral blood mononuclear cells from Crohn’s disease patients secrete spontaneously sHLA-G while those from UC patients and healthy donors do not. Furthermore, after stimulation with LPS, both cells from Crohn’s disease and healthy subjects show sHLA-G production, while this does not happen in UC patients. The different HLA-G expression profiles in UC and Crohn’s disease patients sustain the different aethiopathogenesis at the origin of these two diseases. In particular, the responses to therapies in UC and Crohn’s disease correspond to different sHLA-G secretion levels (52). The immunosuppressant therapy normalizes the production of HLA-G molecules in Crohn’s disease while it starts the release of HLA-G in UC patients. These data confirm the diversity in the behavior of these two pathologies and propose the analysis of sHLA-G levels with the final goal of distinguishing between UC and Crohn’s disease patients and to monitor therapy.

## HLA-G and Rheumatologic Diseases

Rheumatic diseases are inflammatory and autoimmune diseases, which are the second most common cause of disability after musculoskeletal injuries. Rheumatoid arthritis (RA) is an autoimmune disease caused by the immune system attacking synovial cells. A combination of genetic and environmental factors may increase the risk of RA. Gene expression profiles (GEPs) in bone marrow-derived RA mononuclear cells (53) have shown 1,910 down-regulated and 764 up-regulated gene, which include the *HLA-G* gene. Several studies have evaluated the role of *HLA-G* polymorphisms in RA susceptibility without reaching a final common result. The evaluation on 256 RA patients and 356 healthy controls genotyped for the *HLA-G 14bp INS/DEL* polymorphism has reported no differences in allelic and genotypic frequencies and no correlation with disease characteristics (54). The analysis of two SNPs (rs1736936, −1305G/A and rs2735022, −689A/G) in *HLA-G* promoter in the Korean population has not presented any connection to the development of RA (55). The evaluation in a Brazilian cohort documented the implication of 3′ UTR polymorphisms in RA follow-up (56). The authors have observed a significant association of the −*762C* > *T*, −*716T* > *G*, −*689A* > *G*, −*666G* > *T*, −*633G* > *A*, −*486A* > *C*, and −*201G* > *A* (*rs1632946*; *rs2249863*; *rs2735022*; *rs35674592*; *rs1632944*; *rs1736933*; and *rs1233333*) SNPs with the disease. The analysis of 106 patients with juvenile idiopathic arthritis (JIA) has shown an association between JIA female susceptibility and the 14 bp DEL allele. These different associations support the presence of different pathogenic elements between RA and JIA (54). RA (57) and JIA patients present lower serum sHLA-G concentration than in controls (58), with a possible contribution to the chronicity of the inflammation. On the contrary, JIA synovial fluids showed higher sHLA-G levels than controls (SF) (56). Since we have observed that HLA-G molecules are enhanced in synovial fibroblasts from inflamed joints (59) and that high sHLA-G levels correlate with disease activity (57), we may suggest an impaired control of immune reaction at joint, which characterizes JIA disease. The *HLA-G 14bp INS/DEL* polymorphism has also been evaluated as a marker for RA therapy. Methotrexate (MTX), a disease-modifying anti-rheumatic drug (DMARD), induces an increased production of IL-10 in RA patients with a better therapeutic response (60) and is able to enhance HLA-G secretion by peripheral blood mononuclear cells (61). Interestingly, the *14bp DEL/DEL* genotype is increased in RA patients with a good response to MTX therapy (62), with a possible implication in the control of immune activation. It must be underlined, however, that contrasting results have been obtained (63, 64), possibly due to a different dosage of MTX, a different cut-off value for RA therapy response assessment. Scleroderma (SSc) is an autoimmune rheumatic disease of the connective tissue (65). Only SSc patients with a longer survival, lower frequency of vascular cutaneous ulcers, telangiectasias, and inflammatory polyarthralgia present HLA-G molecule expression in skin biopsies (66) suggesting an implication of this molecule on the control of immune response at the skin level.

Systemic lupus erythematosus is a systemic autoimmune disease of the connective tissue that can affect any part of the body. The immune response is mainly characterized by Th2-cell predominance. Rosado and coauthors (67) and Chen and coauthors (68) have shown higher sHLA-G and IL-10 levels in systemic lupus erythematosus (SLE) patients in comparison with healthy controls, while Rizzo and coauthors (69) have observed lower sHLA-G concentrations in SLE patients (70). Interesting, the analysis of monocytes and mature CD83 positive dendritic cells from SLE patients has evidenced a diminished expression of HLA-G in comparison with healthy controls (71), a lower HLA-G expression in response to IL-10 and a lower HLA-G trogocytosis from autologous monocytes compared with controls. Using the SNPs mapping approach, *HLA-G* gene is recognized as a novel independent locus for SLE (72). In particular, *HLA-G 14bp INS/DEL* polymorphism and *HLA-G* +*3142C* > *G* SNP have been analyzed in a SLE population. SLE patients showed a higher frequency of *14bp INS* allele and *14bp INS/INS* genotype (69) and the heterozygote group showed lower systemic lupus erythematosus disease activity index (SLEDAI) indexes than homozygous groups (73). On the contrary, the evaluation of *HLA-G 14bp INS/DEL* polymorphism in a SLE Brazilian population did not present an association (74), while the +*3142G* allele and the +*3142 GG* genotype frequencies were increased among SLE patients as compared with controls (75, 76). These data sustain a possible role of HLA-G expression in modifying SLE condition. Behçet (BD) and Kawasaki diseases are autoimmune vasculitis. The HLA-G*01:01:01 allele is associated with a reduced risk of BD while HLA-G*01:01:02 and G*01:05N alleles are associated with an increased risk of BD (77, 78). Non-synonymous SNP *(*+*755A/C*) of the *HLA-G* gene (rs12722477, G*01:04) is significantly associated with Kawasaki disease (79). These data suggest an influence of *HLA-G* polymorphisms in determining disease risk, possibly affecting HLA-G production and consequently inflammation status.

## HLA-G and Cutaneous Diseases

The skin is characterized by a “skin immune system (SIS),” where immune cells and humoral components support cutaneous inflammation. The deregulation of skin defense mechanisms is evident in a large variety of inflammatory disorders of the skin, such as psoriasis, atopic dermatitis, pemfigo, vitiligo, and systemic sclerosis (80). HLA-G protein is not expressed in the skin from healthy controls (81, 82). Ectopic HLA-G expression has been described in skin pathologies (83–86).

Psoriasis is a chronic inflammatory skin disease with an autoimmune component. Both membrane-bound and soluble HLA-G proteins have been detected in psoriatic skin lesions with the main compound characterized by macrophage lining at the dermo-epidermal junctions (82). The up-regulation of HLA-G molecules by macrophages could represent an attempt to control auto-reactive T cells, induced by activated keratinocytes-derived cytokines/chemokines. HLA-G may prevent keratinocyte destruction by modulating the activity of cytotoxic lymphocytes and promoting the development of Treg cells (87). Interestingly, significantly lower plasma sHLA-G levels have been found in psoriatic patients compared with controls (88), suggesting a difference in systemic HLA-G expression that could be associated with the IL-10 deficiency typical of psoriasis. Psoriasis management can be divided into three main types: topical drugs, light therapy, and systemic medications. Evaluation of therapeutic effects on sHLA-G expression has shown an increase in plasmatic levels of systemic treated patients (efalizumab, cyclosporin A, and acitretin) (88) and a significant association between *HLA-G 14bp DEL* allele and *14bp DEL/DEL* genotype with acitretin clinical outcome (89). We can suppose a possible direct effect of HLA-G in antagonizing systemic T helper 1 activation and with a potential role as a marker of response to acitretin in psoriatic patients.

Pemphigus vulgaris is a blistering disease caused by autoantibodies to desmoglein skin adhesion proteins. Skin tissue sections from pemphigus vulgaris (PV) patients express detectable HLA-G molecules at both transcriptional and translational levels, while control sections present only HLA-G transcription (90). Moreover, the HLA-G *14bp DEL* allele has been observed with higher frequency in PV patients in comparison with controls in a Jewish population (91). These data suggest that HLA-G expression could be a detrimental factor for the development of PV.

## HLA-G and Diabetes

Type 1 and type 2 diabetes present immunologic defects that enhance insulin resistance as a result of genetics sedentary lifestyle, obesity, and other conditions, such as chronic inflammation or infection. It has been shown that higher levels of sHLA-G are frequent in subjects with an impaired glucose metabolism (92). These data suggest a possible implication of HLA-G antigens in the diabetic condition. In fact, SNPs *rs4122198, rs2394186, rs1619379*, and *rs1611133* near the *HLA-G* gene have been associated with type 1 diabetes (93); dendritic cells from type 1 diabetic patients produce lower HLA-G molecules in response to IFN-beta (94) in comparison with control subjects and the *HLA-G 14bp INS-INS* genotype might contribute to the development of high blood pressure in type 2 diabetes (95).

Interestingly, HLA-G has been found in some secretory granules and on the cell surface of primary islet cells induced to secrete insulin (28). On the basis of these data, it could be hypothesized that an impaired HLA-G expression at pancreatic islets could sustain T cell activation and onset of diabetes.

## HLA-G in Multiple Sclerosis

**Figure 3:**
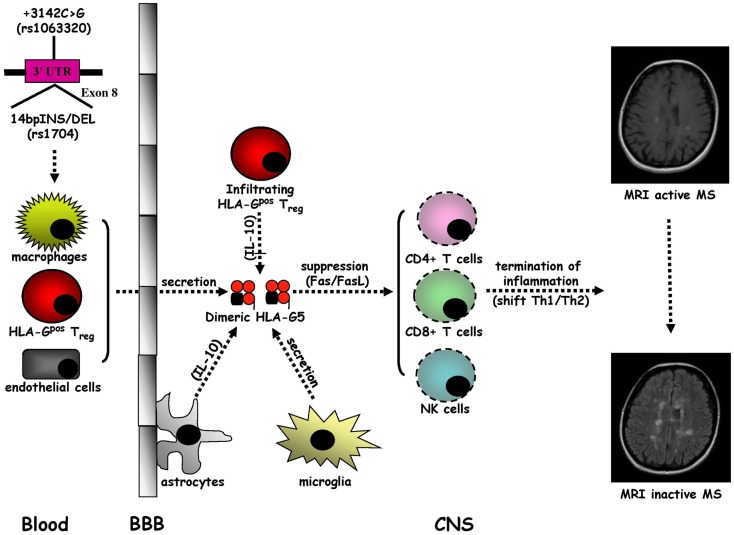
**Intrathecal immune milieu in MS**. The secretion of HLA-G5 in dimeric form by macrophages and HLA-G^pos^ T_reg_ infiltrating the central nervous system (CNS) across the blood–brain barrier (BBB), endothelial cells, and microglia, sustained by a IL-10 release by astrocytes, may promote the suppression of CD4^+^ Th1 cell activity and the apoptotic removal of CD8^+^ T cells and NK cells that favor the formation of an anti-inflammatory intrathecal microenvironment leading to the termination of MS inflammation.

Multiple sclerosis is the prototypic autoimmune disease of the central nervous system (CNS) characterized by chronic inflammatory demyelination and neurodegeneration of unidentified origin (96). Multiple sclerosis (MS) typically occurs in young adults and manifests in women twice as frequently as in men with neurological symptoms and signs, called relapses, which are usually disseminated in space and time (97). About the 80% of MS patients present a disease onset with a relapsing–remitting (RR) form followed by a secondary progressive (SP) course that arises after years, whereas MS starts with a primary progressive (PP) form in approximately the 20% of subjects (98). However, the recent proposed criteria (99) suggest that the coexistence of multi-focal lesions in the periventricular white matter on T2-weighted Magnetic Resonance Imaging (MRI) scans with or without Gadolinium (Gd) enhancement on T1-weighted MRI scans are needed for the diagnosis of MS. Based on epidemiological studies, exposure to an environmental factor, e.g., an infectious agent, in genetically predisposed individuals is currently thought to be crucial for MS pathogenesis (100) in which the traffic into the CNS of activated auto-reactive CD4^+^ T helper 1 (Th1) cells plays a central role (96, 101, 102). The initiation of brain inflammation is due to the activation of microglia by infiltrating CD4^+^ T cells leading to the generation of Th1-mediated immune responses (IL-12/IFN-γ and IL-23/IL-17), while the resolution of neuroinflammation is triggered by astrocytes, which promote anti-inflammatory Th2-polarized responses (IL-10 and TGF-β) and the elimination of infiltrating immune cells through Fas/FasL-dependent apoptosis (96, 101) (Figure 3).

A growing body of evidence indicates that sHLA-G antigens may have a tolerogenic role in MS (102, 103). Cerebrospinal fluid (CSF) detectable sHLA-G has been detected in RRMS patients with higher levels in comparison with other inflammatory neurological disorders (OIND), non-inflammatory neurological disorders (NIND), and controls (104). Furthermore, higher CSF sHLA-G levels have been detected in RRMS without MRI evidence compared to those with MRI active disease. Notably, a positive correlation between CSF concentrations of sHLA-G and IL-10 has been found in MS patients without MRI evidence of active disease. Therefore, CSF levels of sHLA-G may act, together with IL-10, as anti-inflammatory molecules to regulate MS disease activity. The association between elevated CSF sHLA-G levels and clinical and MRI appearance of MS stable disease is supported by the intrathecal synthesis of sHLA-G in MS clinically and MRI inactive patients (105). We have found higher CSF levels of HLA-G5 and not of sHLA-G1 isoforms compared with controls and in presence rather than in absence of MRI Gd enhancing lesions (106) and an as well as inverse correlation between CSF levels of sHLA-G and anti-apoptotic sFas molecules in MS patients without MRI disease activity (107). Collectively, these results suggest a strong correlation between high CSF levels of sHLA-G antigens and the resolution of MS autoimmunity probably related to the anti-inflammatory properties of these molecules. The impact of HLA-G in MS pathogenesis was recently confirmed by other studies, which demonstrated that: (a) Th1 and Th2 cytokine production and CD4^+^ T cell proliferation are suppressed by HLA-G from MS patient peripheral blood monocytes during the first month of treatment with IFN-β (108); (b) MS disease activity during pregnancy may be modulated by tolerogenic properties of sHLA-G since post-partum serum sHLA-G levels are higher in MS patients without clinical attacks (109); and (c) microglia, macrophages, and endothelial cells located within and around MS lesions present a strong immunohistochemical expression of HLA-G and its inhibitory receptors (ILT-2 and ILT-4), with an elevated protein HLA-G expression on cultured human microglial cells after activation with Th1 pro-inflammatory cytokines (110). Meanwhile, a novel subpopulation of naturally occurring CD4^+^ and CD8^+^ regulatory T cells of thymic origin expressing HLA-G (HLA-G^pos^ T_reg)_, has been characterized in MS patients with a suppressive activity through the secretion of HLA-G5 and the shedding of sHLA-G1 (111–113). Overall, these data sustain anti-inflammatory properties of sHLA-G molecules, and in particular HLA-G-5 isoform, which could lead to the remission of MS autoimmunity. Although it has been demonstrated that SNP rs4959039, a SNP in the downstream un-translated region of HLA-G gene is independently associated with MS susceptibility (114), the possible link between *HLA-G* genetic polymorphisms and MS has not been intensively explored (102, 103). Conflicting results have been obtained. Although no association between *HLA-G* gene polymorphism and MS or severity of the disease has been initially found (115), *14bpINS* and −*725G* (rs1233334) alleles have been shown to be related to MS (116). However, a recent study, evaluating the influence of *14bpDEL/INS* and +*3142C* > *G* HLA-G polymorphisms on CSF and serum sHLA-G production, has documented a correlation between HLA-G genetic polymorphisms and sHLA-G concentrations in both CSF and serum (117). These findings indicate that CSF and serum sHLA-G levels in MS could be affected by two main HLA-G polymorphisms. Moreover, preliminary results from our laboratory have demonstrated that, MS patients present dimeric sHLA-G form more frequently than control, in particular in MRI inactive MS patients (unpublished data), suggesting that large amounts of biologically active dimeric sHLA-G form could be released in CSF of MS patients, possibly induced by pharmacological treatment (118). Nevertheless, in a recent study no association was found between serum sHLA-G levels, disability progression, disease MRI activity, and time to conversion from clinically isolated syndrome (CIS) to clinically definite MS (119). These findings suggest that the use of sHLA-G levels in CSF should be taken into consideration as a prognostic marker for monitoring disease conversion, activity, progression, and response to therapy.

## HLA-G Impact in Viral Infections

Even if host immune system present several mechanisms to control viral infections, the viruses have developed several strategies to counteract host immune defenses (120). HLA-G seems to be implicated in viral immune-escape from Natural Killer cells (121).

Human immunodeficiency virus type 1 (HIV-1) up-regulates HLA-G molecules and down-regulates classical HLA-A and -B. Studies have focused on the expression of HLA-G in monocytes, which are relevant as reservoirs of HIV-1, and in lymphocytes, which are more susceptible to infection by HIV-1. Monocytes from HIV-1 seropositive patients express HLA-G (122) with a possible association with antiretroviral therapy (HAART), since patients undergoing HAART present higher levels of HLA-G expression on monocytes in comparison with untreated and healthy subjects (122, 123). T cells obtained from HIV-1 seropositive individuals have been found to express HLA-G at a higher proportion (124) and behave like HLA-G+ Treg. Furthermore, on the basis of *HLA-G* genetics, it would seem that the *HLA-G 14bpINS* and +*3142G* polymorphisms affect the susceptibility to HIV (125) but not mother–child transmission (126) in African population.

Human cytomegalovirus is a herpes virus that persists in the host (127) by means of several strategies to evade the immune system. HLA-G expression is evidenced during viral reactivation in macrophages and astrocytoma cells (35) and the levels of expression on monocytes an in serum is higher during active human cytomegalovirus (HCMV) infection (128). This up-regulation is proposed to be associated with virus-encoded homologs of humanIL-10 (cmvIL-10) (129), which prevents NK cell recognition of infected cells.

There is also evidence to support also a role of HLA-G molecules in susceptibility and outcome of human papilloma virus (HPV) infections. The alleles HLA-G *14bp INS*, +*1537C* (rs12722477), G*01:01, G*01:04, and G*01:06 have been associated with both high-grade squamous intraepithelial lesions and cervical cancer, while *HLA-G 14bp DEL* and +*3142C* alleles have been identified as protective (130–135). These results are in agreement with the low levels of HLA-G5 expression in cervical cancer (136). On the other hand, two researches recognized *HLA-G 14bp DEL* allele and +*3142C* as associated with increased risk of cervical cancer (137, 138), in agreement with increased expression of HLA-G in cervical cancer tissues (139) and with the spontaneous de-methylation of *HLA-G* promoter that allows immune-evasion and the development of precancerous cervical lesions (140). HLA-G has been also implicated in nasal polyposis development in the presence of HPV infection (141). Nasal polyps with HPV11 infection have shown HLA-G expression on epithelial cells, while no HLA-G expression has been observed in HPV negative polyps.

Neurotropic viruses such as herpes simplex virus-1 (HSV-1) and Rabdovirus (RABV) (142) induce the expression and up-regulation of membrane and soluble HLA-G molecules in actively infected neurons with a consequent protection toward host NK cells.

Hepatitis C virus (HCV) and Hepatitis B virus (HBV) seems to induce HLA-G expression to control host immune response (125, 143–148).

On the basis of these results, HLA-G proteins are expressed by virally infected cells as a mechanism to evade host immune control, preventing T cell and NK cell activation. The main challenge would be to block HLA-G up-regulation by viral infection, in order to allow the recognition by immune cells.

## Interaction of HLA-G Molecules with Other HLA-Ib Molecules

Other HLA-Ib molecules have been identified: HLA-E and HLA-F (149, 150) characterized by a low genetic diversity as well as by a particular expression pattern, structural organization and functional profile.

Similar to HLA-G, HLA-E forms a complex with β2-microglobulin. HLA-E is known to play an important role as immune-modulator during pregnancy and transplantation (151), inhibiting immune responses by its interaction with CD8^+^ T cell receptors (TCRs) (152) and with the CD94/NKG2A inhibitory receptors of NK cells (153). Meanwhile, this molecule may present non-self antigens activating immune response (154).

Similar to other HLA molecules, HLA-F can form a complex with beta2 microglobuli and three splicing variants have been described. While the presence of HLA-G and HLA-E has been recently correlated with physiological and pathological conditions, the clinic-pathological significance of HLA-F is limited. HLA-F is expressed by peripheral blood B cells upon activation (155) and is detected in embryonic tissues, including the extravillous trophoblasts invading maternal deciduas, and in spermatozoids (156, 157) and in the serum of patients affected by tumors (158).

Only few data are available on the interaction of HLA-G molecules with the other HLA-Ib antigens. In physiological conditions, HLA-G molecules interact with HLA-E and co-operate to inhibit NK cells, mainly at feto-maternal interface, via interaction with ILT-2 and CD94/NKG2A, respectively (159). In pathological condition, the interaction between these two molecules facilitates the escape of tumor cells from NK cell recognition (160). In MS, HLA-G and HLA-E molecules are expressed by resident CNS cells and interact with NK cell and cytotoxic lymphocytes (161). HLA-G, -E, and -F expression by trophoblasts correlates with the protection of the fetus from destruction by the maternal immune system, suggesting a co-operation for fetal tissue preservation.

## Conclusion

This review aims to focus on the key role of HLA-G molecules in autoimmune diseases and viral infections. The data herein summarized suggest that HLA-G may have a crucial role in the creation of an impaired immune response that characterizes these pathological conditions.

In fact, it appears even more evident that HLA-G proteins are involved in the regulation of the immune system during autoimmunity, such as gastrointestinal, skin, rheumatic and neurological diseases and in the immune-escape mechanisms during viral infections.

Here, we have reviewed a series of experimental and epidemiological studies that support the direct influence of HLA-G proteins on the balance of immune settings. On this basis, understanding the function of HLA-G in these disorders could help in the identification of new approaches to control HLA-G production.

For example, it is interesting to note that inflammatory cutaneous diseases present a disproportional expression of HLA-G molecules with respect to controls and that this could generate autoimmunity. Thus it appears that down/over-expression of HLA-G may not only act as an immunosuppressive and beneficial molecule but may also sustain an unbalanced immune stimulation and autoimmunity. With reference to bowel diseases especially, it appears clear that the different HLA-G expression levels could help in the differential diagnosis and consequently in the choice of appropriate treatment.

Furthermore, several studies have evidenced the possible role of sHLA-G antigens as a tolerogenic molecules in MS since their intrathecal production is associated with disease remission. It is of extreme importance to evaluate the role of HLA-G antigens in MS pathogenesis, in particular if they are implicated in disease progression or if they represent an indirect manifestation of MS inflammation of CNS. Still to be clarified are the functional differences between HLA-G5 and sHLA-G1, and whether dimers and monomers exert a different function in MS inflammatory disease activity. As far as viral infections are concerned, HLA-G could be considered a target for anti-viral treatment, so increased knowledge in this field could contribute to identifying different therapeutic strategies.

Collectively, the results emerging from the literature confirm the importance of the HLA-G molecule in the pathogenesis and progression of immune-based diseases and infections, underlining the relevance of its investigation with the aim to developing new therapeutic strategies and clinical markers. Meanwhile, the analysis of the interactions between HLA-G and other HLA-Ib molecules may be useful to understand the mechanisms for the creation of immune-suppressive microenvironments.

## Conflict of Interest Statement

The authors declare that the research was conducted in the absence of any commercial or financial relationships that could be construed as a potential conflict of interest.
